# Association between dietary patterns and renal function in a cross-sectional study using baseline data from the ELSA-Brasil cohort

**DOI:** 10.1590/1414-431X202010230

**Published:** 2020-10-30

**Authors:** G.B. Silva, S.D.S. Fraser, A.K.M. Néri, R.M.F. Xavier, R.M.S. Mota, A.A. Lopes, J.G. Mill, S.M. Barreto, V.C. Luft, D. Chor, C.A.S.T. Santos, P.A. Lotufo, S.M.A. Matos

**Affiliations:** 1 Instituto de Saúde Coletiva, Universidade Federal da Bahia, Salvador, BA, Brasil; 2 Programa de Pós-Graduação em Saúde Coletiva, Centro de Ciências da Saúde, Universidade de Fortaleza, Fortaleza, CE, Brasil; 3 School of Primary Care, Population Sciences and Medical Education, Faculty of Medicine, University of Southampton, Southampton, United Kingdom; 4 Departamento de Estatística e Matemática Aplicada, Centro de Ciências, Universidade Federal do Ceará, Fortaleza, CE, Brasil; 5 Departamento de Ciências Fisiológicas, Centro de Ciências da Saúde, Universidade Federal do Espírito Santo, Vitória, ES, Brasil; 6 Departamento de Medicina Preventiva e Social, Faculdade de Medicina, Universidade Federal de Minas Gerais, Belo Horizonte, MG, Brasil; 7 Programa de Pós-Graduação em Epidemiologia, Faculdade de Medicina, Universidade Federal do Rio Grande do Sul, Porto Alegre, RS, Brasil; 8 Departamento de Epidemiologia e Métodos Quantitativos em Saúde, Escola Nacional de Saúde Pública, Fundação Oswaldo Cruz, Rio de Janeiro, RJ, Brasil; 9 Laboratório de Epidemiologia Molecular e Bioestatísticas, Centro de Pesquisa Gonçalo Moniz, Fundação Oswaldo Cruz, Salvador, BA, Brasil; 10 Divisão de Medicina Interna, Centro de Pesquisa Clínica e Epidemiológica, Hospital Universitário, Universidade de São Paulo, São Paulo, SP, Brasil

**Keywords:** Dietary pattern, Feeding behavior, Kidney diseases, Albuminuria, Glomerular filtration rate

## Abstract

Previous analyses of the Brazilian Longitudinal Study of Adult Health (ELSA-Brasil) identified four main dietary patterns (DP). The aim of this study was to explore the association between the previously defined DP and renal function (RF). A cross-sectional study using the ELSA-Brasil baseline data was carried out. DP (“traditional”, “fruits and vegetables”, “bakery”, and “low sugar/low fat), metabolic syndrome (MS) using the Joint Interim Statement criteria, microalbuminuria (MA), and glomerular filtration rate (eGFR) through the CKD-EPI equation were evaluated. Abnormal RF was defined as eGFR<60 mL·min^-1^·(1.73 m^2^)^-1^ and MA≥3.0 mg/dL. Factors associated with RF were determined and mediation analysis was performed to investigate the association between DP, MS, and RF. A total of 15,105 participants were recruited, with a mean age of 52±9 years; 8,134 participants (54%) were females. The mediation analysis identified indirect associations between “bakery” and “fruits and vegetables”, and both were associated with decreased eGFR and albuminuria in both genders, compared with “traditional” and “low sugar/low fat” patterns in the general population. There was a direct association of the “bakery” pattern with MA in men (OR: 1.17, 95%CI: 1.92-1.48). The “fruits and vegetables” pattern also showed a direct association with reduced eGFR in women (OR: 1.65, 95%CI: 1.28-2.12), although there was no significance after adjustment. The “fruits and vegetables” and “bakery” DPs were associated with renal dysfunction. The only independent, direct association was between “bakery” DP and MA in men, raising concerns about DP and renal damage in men.

## Introduction

Despite the recession that started after 2015 due to the economic changes that occurred after the 2008-2009 global financial crisis, in the past decades, the socioeconomic progress of Brazil has been remarkable and internationally noted. The country has been successful in reducing income inequalities and poverty, although it is still one of the most unequal societies worldwide (1,2).

The emerging better economy of Brazil has had an important impact on Public Health, including increased life expectancy and reduction in infant mortality, however, it has increased the rates of chronic, non-communicable diseases, obesity, hypertension, and diabetes, all risk factors for chronic kidney disease (CKD) (3,4). The population's dietary habits have also changed dramatically over recent decades, unfortunately for the worst. A recent, large population-based study carried out in Brazil identified a higher frequency of unhealthy eating habits in adults, with low regular consumption of fruits and vegetables and a high consumption of soft drinks and red meat (5).

All of these changes could have contributed to the high burden of CKD in Brazil, which is growing, and has been increasingly recognized as a global Public Health concern (6). CKD affects approximately 8.2% of the Brazilian population and is currently among the top 10 causes of death in Brazil, representing 2.62% of all-cause mortality in this country (7). From 2009 to 2018, the estimated global prevalence of patients on chronic dialysis went from 405 to 640 per million people, corresponding to an absolute increase of 58%, with an average increase of 6.4% per year (8). This growing number of patients is unsustainable for the health system, and preventive, upstream public health measures, such as those influencing diet and physical activity, are urgently needed to tackle the growing problem.

Previous studies in other settings have identified associations between some dietary patterns and CKD, and dietary changes have been proposed as an important approach to slow CKD progression (9–12). “Healthy” dietary habits, including sodium restriction, fruits and vegetables consumption, and increased water intake may slow early CKD progression, as suggested in previous studies (12). A recent study showed an association between sodium restriction and lower levels of albuminuria in Chinese adults from a region where hypertension is highly prevalent (13). A large cohort conducted in the USA, with more than 500,000 adults, showed that healthy diets based on scores (Alternate Healthy Eating Index, Healthy Eating Index, Mediterranean Diet Score, and Dietary Approaches to Stop Hypertension - DASH) were associated with a reduced risk for CKD (14). Another cohort study, also in the USA, but with a smaller sample (1,534), found a significant association between “poor accordance to a DASH dietary pattern” and glomerular filtration rate decline in patients with hypertension (15). Increased intake of fruits and vegetables and limiting alcohol consumption were associated with a lower risk of requiring dialysis or renal function worsening in patients with CKD stages 3-4 (16).

In Brazil, there have been few studies on this subject. A recent study comparing patients with CKD to those without CKD showed that CKD patients tend to have a healthier diet, mainly based on healthcare advice (17). In the largest cohort study performed in Brazil, the Longitudinal Study of Adult Health (ELSA-Brasil), cluster analyses of dietary behaviors identified four dominant dietary patterns in the population according to the frequency of consumption of key components: traditional, fruits and vegetables, bakery, and low sugar/low fat pattern (18). The association between these dominant dietary patterns and the presence of CKD has not been explored.

Therefore, the aim of this study was to explore the association between these predominant dietary patterns in Brazil and renal function using baseline data from the ELSA-Brasil cohort study. We hypothesized that dietary patterns can be associated with renal function, and this association can be influenced by metabolic syndrome (MS) components (elevated waist circumference, hypertension, diabetes, dyslipidemia), through a mediation model of association.

## Material and Methods

### Study design

This multicenter prospective cohort study, ELSA-Brasil, began in 2008 and provided data for the present investigation. This study is described in detail elsewhere (19,20). Briefly, the ELSA-Brasil was designed to understand the development and progression of clinical and subclinical chronic diseases. At baseline, ELSA-Brasil enrolled 15,105 active or retired employees from 6 large Brazilian cities (Belo Horizonte, Porto Alegre, Rio de Janeiro, Salvador, Sao Paulo, and Vitoria), located in the Northeast, Southeast, and South regions of Brazil (19,20). The ELSA-Brasil study was approved by the ethics committee of each participating institution, and participants gave written informed consent before enrollment in the study. The present investigation, a cross-sectional study using baseline data from the ELSA-Brasil population, explored the associations of dietary patterns and renal function, considering MS components as possible mediators of this association.

### Study population

The study population is comprised of adults aged 35 to 74 years, living in Brazil, all civil servants from public universities and research institutes. This population was chosen aiming to minimize losses to follow-up related to geographical mobility. Exclusion criteria included severe cognitive or communication impairment, intention to quit work at the institution in the near future for reasons not related to retirement, and, if retired, residence outside the corresponding metropolitan area (20). A total of 14,921 participants had the glomerular filtration rate (GFR) estimated and 14,640 had albuminuria measured and were then included in the analysis.

### Measurements

Data were obtained on age, gender, ethnicity, level of schooling (elementary school or lower, high school, university or higher - based on employees' registers), anthropometry (weight, height, body mass index - BMI, and waist circumference), using standardized techniques (19), dietary patterns, MS components, serum creatinine, microalbuminuria (MA), and estimated glomerular filtration rate (eGFR).

### Dietary assessment

Food consumption was measured through a previously validated food frequency questionnaire (21) and then the number of food frequency categories was redefined using multiple correspondence analysis (MCA) in each food group and cluster analysis to identify the dietary patterns. As shown in Table 1, the four main dietary patterns previously defined in ELSA-Brasil cohort were based on the presence or absence of consumption of the so-called key foods: traditional pattern (beans, rice, refined cereals), fruits and vegetables pattern (green vegetables, fruits, little red meat), bakery pattern (refined cereals, bread, cookies, fried chicken, dairy products), and low sugar/low fat pattern (wholegrain cereals, skim milk, sugar-free or soy-based beverages) (18).

**Table 1:** Dietary patterns in the ELSA-Brasil cohort.

Dietary pattern	Frequency of food consumption			
	Daily consumption	Weekly consumption	Daily or weekly consumption	Absence of consumption
Traditional	Semi-skimmed milk and dairy products	Milk and dairy products semi-skimmed; refined cereals; white meats; fruits and vegetables in general	More energy-dense confectionery; processed red meats and eggs; nuts; beans; other legumes; beverages with sweeteners and beverages with sugar	Fast food
Fruits and vegetables	Pumpkin and cabbage; white cheese and semi-skimmed milk; light green vegetables; popular and less popular fruit; pasta and instant pasta; raw green vegetables and chicken breast		Coconut water	Processed red meats and eggs; beans; refined cereals and confectionery
Bakery products	Broiled or fried chicken; whole milk and dairy products; refined cereals; potato and flours			Nuts and other grains; lentils and other legumes; fruits and vegetables
Low sugar/low fat	Sugar-free and soy-based beverages; oats		Skimmed milk; whole-grain rice and less energy-dense confectionery	

We decided to conduct an analysis of the general sample and a separate analysis by gender (male and female) to investigate possible associations between these dietary patterns and renal function, as it has already been demonstrated that there are significant differences in the prevalence of dietary patterns between men and women in this cohort's baseline analysis (the “traditional” and “bakery” patterns were more frequent in men, while “fruits and vegetables” and “low sugar/low fat” were more frequent in women) (18).

### Renal function

Renal function was assessed by the eGFR, estimated through the CKD-EPI equation, with one blood sample collection (22), and by MA, using the albumin-creatinine ratio (ACR) with a 12-h urine collection. We considered abnormal renal function as eGFR<60 mL·min^-1^·(1.73 m^2^)^-1^ and an abnormal MA as ≥30mg/dL (23). The kinetic Jaffé's method was used to measure urinary creatinine levels (Advia 1200 Siemens, USA), and the immunochemical assay was used to measure urinary albumin (BN II Nephelometer Siemens Dade Behring, USA). All analyses were performed at the University of Sao Paulo.

### Other measurements

#### Covariates

The MS components were defined as: elevated waist circumference (≥102 cm in men and ≥88 cm in women), arterial hypertension (AH, previous diagnosis or systolic blood pressure ≥140 mmHg and/or diastolic blood pressure ≥90 mmHg), diabetes mellitus (DM, fasting glucose ≥100 mg/dL), high-density lipoprotein (HDL) <40mg/dL in males and <50 mg/dL in females, and triglycerides ≥150 mg/dL, according to the standard guidelines (24). Normal weight was considered as BMI within the range of 18.5 to <25 kg/m^2^, underweight as BMI <18.5 kg/m^2^, overweight as BMI between 25 and <30 kg/m^2^, and obesity as BMI ≥30 kg/m^2^ (25). Other measurements were: age (classified in age groups: 35-44, 45-54, 55-64, 65-74), gender, ethnicity (black, white, “mixed-race” - which is the typical Brazilian mixture of races, Asian, and the Brazilian indigenous population), level of schooling (elementary, high school, university), time of DM, and time of AH, with both variables being reported only by those individuals who already knew themselves to be diabetic and/or hypertensive, respectively, in the cohort baseline. All these other measurements were assessed through the previously described self-reported questionnaires (26).

### Statistical analysis

All statistical analyses were conducted using STATA^®^ software (version 12; StataCorp, USA), and for the descriptive statistics, continuous variables are reported as means±SD. The Student's *t*-test for independent samples was used to test the difference between the groups according to eGRF and MA. Pearson's chi-squared test or Fisher's exact test was used to test differences in proportions between the eGRF and MA groups. Multivariable odds ratios (adjusting for age, gender, ethnicity, and level of schooling) with 95% confidence intervals were derived using logistic regression. Subgroup analyses with diabetic patients and hypertensive individuals by gender were also performed.

The mediation analysis hypothesizes that an independent variable “X” affects a dependent variable “Y” through one or more potential intervening variables, or “M” mediators (27). This model was conducted to investigate the association between dietary patterns, MS, and renal function. Therefore, in the present study, we investigated if the dietary patterns (“X”) are associated with renal dysfunction (“Y”), and if this association is mediated by intervening variables, which could be components of the MS (“M”). A P value <0.05 was considered statistically significant.

## Results

The participants' mean age was 52±9 years, 8134 (54%) were females, 6078 (41%) were overweight, 3455 (23%) were obese, 2967 (20%) had DM, 5402 (36%) had systemic arterial hypertension (SAH), 691 (5%) had eGFR<60 mL·min^-1^·(1.73 m^2^)^-1^, and 734 (5%) had albuminuria ≥30 mg/g of Cr (Supplementary Table S1). Reduced eGFR was observed in 354 men (5.2%) and 337 women (4.1%) (P=0.002), while MA was found in 406 men (6.0%) and 328 women (4.1%) (P<0.001).

**Figure 1:**
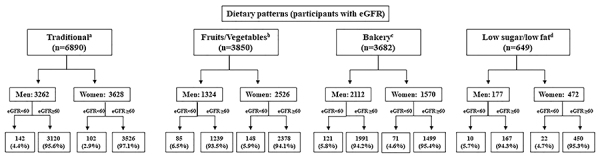
Distribution of dietary patterns according to estimated glomerular filtration rate (eGFR) in men and women. ^a^P=0.0007, ^b^P=0.52, ^c^P=0.11, ^d^P=0.68 (Fisher's exact test).

The analysis of the distribution of dietary patterns identified the traditional or mixed Brazilian diet in 45.7%, fruits and vegetables pattern in 25.5%, bakery pattern in 24.4%, and low sugar/low fat pattern in 4.3% of the cohort participants. The most frequent dietary pattern among the female participants with both normal and reduced eGFR was “fruits and vegetables” (30.4 and 43.9%, respectively), while the most frequent dietary pattern among the male participants was the “traditional” one (40.1% of those with reduced eGFR and 48.4% of those with normal eGFR) followed by the “bakery” pattern (34.1 and 30.9%) (Supplementary Table S1). Similar patterns were observed according to MA levels, but among women with MA, the “traditional” pattern, identified in 40.2% (44% of those without MA), was followed by “fruits and vegetables” in 32% (30.9% of those without MA) (Supplementary Table S2). Analyzing the distribution of eGFR according to the dietary pattern, the highest prevalence of reduced eGFR was found in men (with all dietary patterns), of which the highest were identified in those with the “fruits and vegetables” and “bakery” patterns (6.5 and 5.8% respectively; Figure 1). The only statistically significant difference between men and women was related to the traditional pattern, in which reduced eGFR prevalence was higher among men (4.4 *vs* 2.9%; Figure 1).

Statistically significant univariate associations in the general population were identified between measures of kidney function and damage (reduced eGFR and MA) and age, male gender, presence of AH, DM, hypertriglyceridemia, overweight and obesity, waist circumference, MS, and dietary patterns (Supplementary Tables S1 and S2). The univariate analysis performed in the subgroup of diabetic patients showed an increased association of MA with the “bakery” pattern (P=0.019), with no significant association of this diet with either male or female gender. In the subgroup of hypertensive patients, there was also an association of MA with the “bakery” pattern (P<0.001), which was also identified only in men (P=0.017). There was no significant association between eGFR and dietary patterns in any of the subgroups.

Logistic regression performed in the diabetic subgroup revealed a reduced GFR association with excessive alcohol consumption (OR 0.26; 95%CI 0.08-0.8; P=0.019), presence of AH (OR 2.93; 95%CI 1.70-5.05; P<0.001), older age (OR 1.12; 95%CI 1.09-1.16; P<0.001), and DM time (OR 1.02; 95%CI 1.01-1.04; P=0.010), in addition to an association between increased MA with lower levels of schooling (incomplete elementary school: OR 1.95; 95%CI 1.25-3.05; P=0.003), hypertriglyceridemia (OR 2.02; 95%CI 1.49-2,73; P<0.001), and DM time (OR 1.04; 95%CI 1.03-1.06; P<0.001). In the hypertensive individuals' subgroup, there was an association of reduced GFR with lower levels of schooling (complete elementary school: OR 1.46; 95%CI 1.07-2.00; P=0.018), low HDL levels (OR 1.52; 95%CI 1.21-1.92; P<0.001), presence of DM (OR 1.40; 95%CI 1.15-1.71; P=0.001), and older age (OR 1.12; 95%CI 1.10-1.13; P<0.001), and also MA with low levels of schooling (incomplete elementary school: OR 1.8; 95%CI 1.27-2.53; P=0.001), presence of DM (OR 2.82; 95%CI 2.27-3, 5; P<0.001), and AH time (OR 1.02; 95%CI 1.01-1.03; P=0.002).

**Figure 2:**
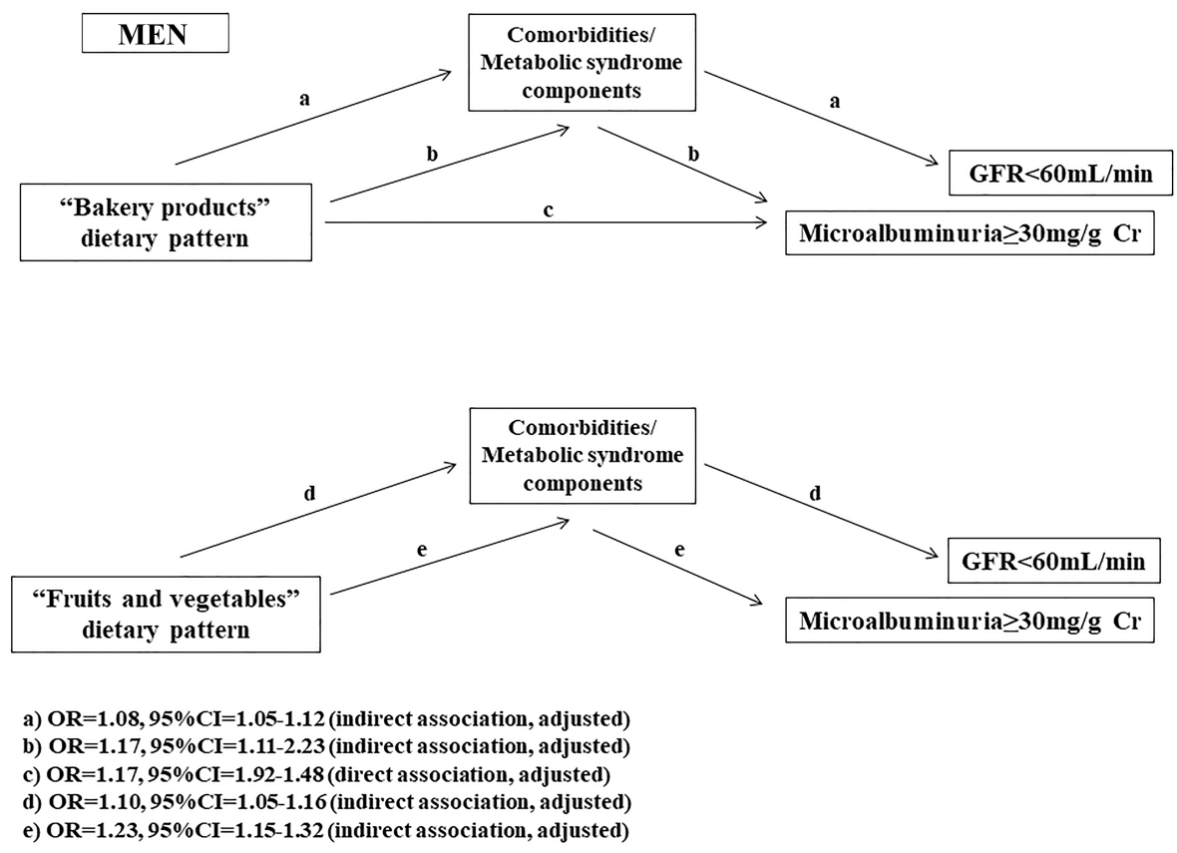
Results of mediation analysis. Association between dietary patterns and renal function mediated by metabolic syndrome for men (analysis adjusted for age, gender, ethnicity, and level of schooling). Comorbidities/metabolic syndrome components: elevated waist circumference, hypertension, diabetes, dyslipidemia, and obesity.

**Figure 3:**
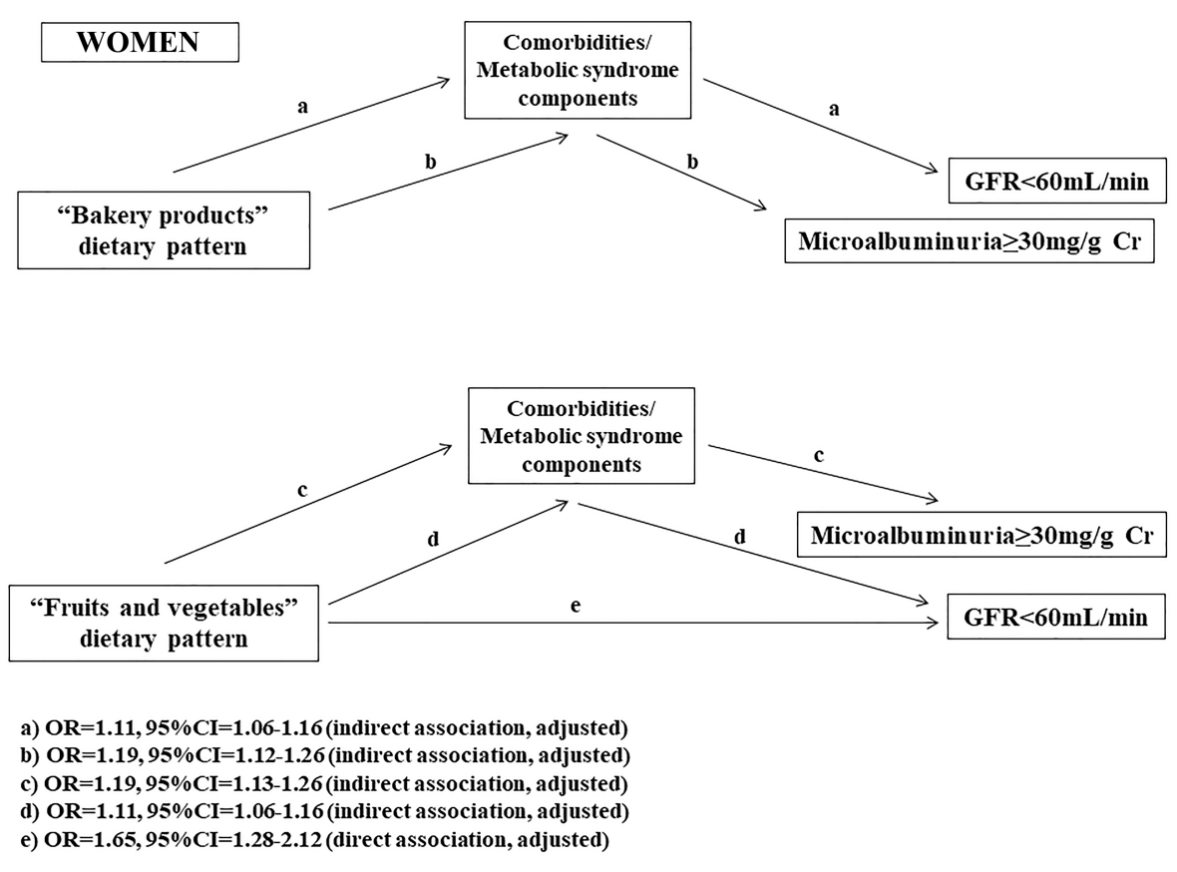
Results of mediation analysis. Association between dietary patterns and renal function mediated by metabolic syndrome for women (analysis adjusted for age, gender, ethnicity, and level of schooling). Comorbidities/metabolic syndrome components: elevated waist circumference, hypertension, diabetes, dyslipidemia, and obesity.

The mediation analysis showed that the “bakery” pattern had a total significant association with renal function for both men and women, but a direct association only in men regarding MA. The “fruits and vegetables” pattern also showed a significant association with renal function, with total and direct associations with reduced eGFR only in women (Supplementary Table S3). In the proposed models shown in Figures 2 and 3, the MS components had a direct association with renal dysfunction (reduced eGFR and MA). The “bakery” pattern had a direct association with MA in men (Figure 2), while the “fruits and vegetables” pattern had a direct association with reduced eGFR in women (Figure 3). Overall, dietary patterns showed an indirect association with renal dysfunction, as shown Supplementary Table S3.

## Discussion

This is the first study in Brazil and one of the largest in the world to investigate the association between dietary patterns and renal function in a large cohort of adults, and important associations were found. Using pre-defined predominant dietary patterns derived from the cluster analysis (18), in the general population, the “bakery” and “fruits and vegetables” patterns showed significant associations with reduced eGFR and MA, with important differences between genders. We found a significant direct association between the “bakery” pattern and MA in men, while a direct association was found between the “fruits and vegetables” pattern and reduced eGFR in women. So far, it is not possible to ensure that there is a cause-and-consequence relationship linked to our findings due to the cross-sectional design of the present study, but further analysis will be performed when prospective data from the ELSA-Brasil cohort are available. It is possible that participants adopting a “non-healthy” diet, such as “bakery” products, have a higher incidence of renal dysfunction or a more accelerated CKD progression. Other factors may appear in the new analysis, which may play a role in causing or worsening CKD in this population.

The study of dietary patterns is important because it represents the groups of food an individual commonly and regularly consumes (18,28). In a cross-sectional study with women from Ireland, a “non-healthy” diet (“Western-like”) was significantly associated with CKD compared to the group adopting healthy dietary patterns (“DASH” or “Mediterranean” patterns) (29). “Healthier” diets, such as DASH and the Mediterranean diets, are associated with lower cardiovascular risk and mortality (30).

The ELSA-Brasil cohort allowed the identification of four dietary patterns in Brazilian adults, which may reflect the dietary habits in Brazil. Detailed characteristics of these patterns have been previously published (18) and are specified in the methods sections of this manuscript. These patterns may reflect the most common dietary patterns adopted by Brazilian people, although this is not a population-based study. The association between these patterns and renal function have not been investigated in the ELSA-Brasil cohort or in other studies carried out in Brazil. Reduced eGFR and MA were more frequent in participants that adopted the “fruits and vegetables” and “bakery” products patterns in the general population, and no significant differences were observed between men and women regarding the associations of these patterns with renal function.

Although it was shown only in the univariate analysis, in diabetic patients, the subgroup analysis revealed an association between the “bakery” dietary pattern and MA, with no gender differences. There was also an association between the “bakery” pattern and MA in hypertensive patients, with this association being also identified in men. No association was shown between dietary patterns and eGFR. A Chinese study that evaluated the association between dietary patterns and kidney function in diabetic patients showed that only a “healthier diet”, rich in fish and vegetables, had a significant association with better parameters of kidney function (10 [Bibr B11]). The findings of a study carried out with hypertensive patients and moderate CKD found that a low acceptance of a healthy diet (DASH diet) was associated with an increased risk of end-stage CKD (31). Our findings are in agreement with both above-mentioned studies.

For men and women in the general population, mediation analysis showed that the “bakery” pattern had a total and indirect association with reduced eGFR. For men, the “bakery” pattern showed a total, indirect and direct association with MA, which means an association independent of the MS, while for women, this pattern showed an indirect association only, which means a mediation by MS. These findings illustrated a possible causal role of this “non-healthy” diet consisting predominantly of bakery products, being a probable risk factor for CKD, and may reflect the adoption of healthier dietary habits by those with diagnosed MS components (diabetes, hypertension, dyslipidemia, and obesity) or CKD. However, we could not identify the direction of causality in the present study due to the cross-sectional nature of the analysis.

The “fruits and vegetables” pattern showed an indirect association only, with reduced eGFR for men, which means that MS significantly influenced this association, while for women, this pattern showed a total, indirect, and direct association. These gender differences may be due to the fact that, even when they have MS and other risk factors for CKD, men adopt a healthier diet less frequently than women. Women take better care of their health compared to men and also show a higher frequency of healthcare seeking, which could lead to them being more often diagnosed with health problems and, as a consequence, adopt healthier diets. In general, women seek healthcare professionals more frequently than men (32,33). Therefore, the association between the “fruits and vegetables” dietary pattern and renal dysfunction may derive from reverse causality, which is an important issue to be discussed in epidemiological studies (34).

Other studies suggest that some dietary patterns are associated with kidney function. A diet characterized by high amount of grains, fruits, vegetables, and dairy food, with low-fat products was associated with lower MA levels (35). In a cohort of 3,121 women in the USA, three dietary patterns were identified: a “prudent” diet (fruits, vegetables, white meat, and grains), the “Western diet” (red meat, processed meat, saturated fat, and sweets), and the DASH diet (fruits and vegetables, whole grains, other mineral-rich foods, low saturated fats, and low-sodium) (36). The Western diet was associated with MA, while the DASH dietary pattern was associated with lower renal function decline and lower frequency of MA (37,38). The “prudent” diet described in that study is similar to the “fruits and vegetables” diet found in our study. Other studies also showed a significant association between the DASH diet and low CKD risk (38,39). In the present study, we can also propose the fact that this is a cohort of individuals with higher socio-economic level, as they are civil servants from Brazilian universities, as a possible cause for the association we found between a healthier diet (“fruits and vegetables”) and renal dysfunction, in addition to the above-mentioned reasons,. Therefore, this cohort consists of individuals who have greater access to adequate medical guidance and care and who, because they have a higher educational level, probably also have a greater capacity to put into practice healthier dietary standards advised by health teams, aiming to treat their previously diagnosed kidney disorders. The association between a “healthy” diet and renal dysfunction has also been previously demonstrated (17).

The study of different dietary patterns has resulted in recommendations for healthier diets, such as the DASH and Mediterranean diets. The DASH pattern has been found to be associated with a decreased risk of GFR decline. The Western diet (higher intake of red meat, saturated fat, and sweets) was found to be associated with a higher risk for developing MA (38). Governments and authorities have proposed some nutritional guidelines for both health professionals and the general population in an attempt to achieve a healthier lifestyle and thus prevent chronic diseases. The World Health Organization has recently published guidelines regarding a healthy diet, emphasizing its benefits for preventing non-communicable diseases, including hypertension (40). The most recent Brazilian dietary guide for the general population adopts this practice and stimulates the consumption of natural foods, salt intake reduction, and avoidance of ultra-processed foods (41). Our study may highlight some novel aspects of the dietary patterns in Brazil and CKD risk, which could be included in the next guidelines aiming at prevention and increased awareness of the potentially harmful dietary components.

This is a large Brazilian cohort with accurate data on diet, which may provide an insight of the dietary patterns adopted in Brazil. We had a high proportion of participants in this cohort who had eGFR and albuminuria measured, and the CKD-EPI equation was used, which provides a very accurate estimation of renal function. This is the first study in our region to show an association between dietary patterns and renal function, and one of the largest in the world.

In summary, renal function in this analysis was associated with dietary patterns, predominantly the “fruits and vegetables” and “bakery” products patterns. The analysis of cause and effect of diet on renal function was not possible due to the cross-sectional design that was used, since we only had baseline data from the ELSA-Brasil cohort. The “fruits and vegetables” and “bakery” products dietary patterns were associated with renal dysfunction, varying with gender. A “non-healthy” dietary pattern (“bakery”) possibly had a negative impact on renal function, while a “healthy” pattern (“fruits and vegetables”) may be associated to renal dysfunction in our analysis as this pattern was often prescribed for people at risk for CKD, as seen in a previous cross-sectional study (17). This “healthy” diet option seemed to be more frequently adopted by women, who are known to seek medical help more often than men.

### Study limitations

The main study limitation was the cross-sectional design; therefore, it was not possible to demonstrate a causal association between dietary patterns and renal function until now; also, findings due to reverse epidemiology can be observed. Further studies in this cohort will provide some evidence on causal associations between diet and renal function and may rule out the reverse epidemiology findings.

Another limitation that we should point out is that the ELSA-Brasil study is not a population-based study, because this cohort evaluates civil servants in Brazil, people who usually have a higher than average socioeconomic level. Therefore, the findings may not represent the Brazilian population as a whole. In addition, the single-sample estimation of renal function (eGFR) limits the accuracy for the diagnosis of CKD or even rules out any transitory or acute renal function decrease, which can bias the results. However, the findings reported in the present study are of great importance because they showed, in an unprecedented way, an important correlation between dietary patterns and renal dysfunction in Brazil.

## Supplementary Material

Click here to view [pdf].
